# FACILITATORS AND BARRIERS IN NAVIGATING COMMUNITY AND HEALTH CARE SYSTEM AMONG INFORMAL CAREGIVERS OF OLDER ADULTS

**DOI:** 10.1093/geroni/igae098.3531

**Published:** 2024-12-31

**Authors:** Boah Kim, Andrew Wister, Barbara Mitchell, Lun Li, Laura Kadowaki

**Affiliations:** Simon Fraser University, Vancouver, British Columbia, Canada; Simon Fraser University, Vancouver, British Columbia, Canada; Simon Fraser University, Vancouver, British Columbia, Canada; MacEwan University, Edmonton, Alberta, Canada; Simon Fraser University, Vancouver, British Columbia, Canada

## Abstract

Informal caregivers are fulfilling important support roles for older adults who tend to require a greater range of services while navigating these community and healthcare systems to fulfill their growing complex needs. Yet, there is sparse knowledge on the most significant facilitators and barriers to effective system navigation (SN) among caregivers. This paper aims to fill these gaps through an investigation of the key factors affecting SN difficulties among informal caregivers of older adults. The Behavioural-Ecological Framework of Healthcare Access and Navigation (BEAN) model is used to frame the study. Using the General Social Survey (GSS) on Caregiving and Care Receiving 2018, a total of 2,733 informal caregivers whose primary care receivers were aged 65 or older were analyzed. Hierarchical logistic regression showed that the probability of reporting SN difficulties was lower for caregivers with social capital/cohesion compared to those without social capital/cohesion. In comparison, the probability of reporting SN difficulties was higher among caregivers with caregiving supports and among caregivers whose care receivers use a higher amount of formal health services. Several sociodemographic covariates (e.g., sex, education level, self-rated stress) were also identified. Given the significance of the social capital and utilization factors, as well as several key covariates as facilitators/barriers in SN among informal caregivers, our findings support certain aspects of the BEAN model. To address the challenges and to fill the care gaps on SN among informal caregivers, there is a need to implement coordinated schemes and health policy for older adults and their caregivers.

